# Editorial Note: Machine learning model for predicting the optimal depth of tracheal tube insertion in pediatric patients: A retrospective cohort study

**DOI:** 10.1371/journal.pone.0325971

**Published:** 2025-06-06

**Authors:** The PLOS One Editors

The *PLOS One* Editors issue this Editorial Note to inform readers that after this article’s [1] publication, we identified potential competing interests between the authors and one or more of the peer reviewers. PLOS reviewed this matter and concluded that the article’s publication is supported based on expert input that was received during pre-publication peer review, including from individuals for whom there are no known competing interest issues.
